# Incidence and risk factors of acute kidney injury in COVID-19 patients with and without acute respiratory distress syndrome (ARDS) during the first wave of COVID-19: a systematic review and Meta-Analysis

**DOI:** 10.1080/0886022X.2021.2011747

**Published:** 2021-12-09

**Authors:** Faraj K. Alenezi, Mohammed A. Almeshari, Rahul Mahida, Mansoor N. Bangash, David R. Thickett, Jaimin M. Patel

**Affiliations:** a Birmingham Acute Care Group, Institute of Inflammation and Ageing, College of Medical and Dental Sciences, University of Birmingham, Birmingham, UK; b Anaesthesia Technology Department, College of Applied Medical Sciences, King Saud Bin Abdul-Aziz University for Health Sciences, Riyadh, Saudi Arabia; c Rehabilitation Health Sciences Department, College of Applied Medical Sciences, King Saud University, Riyadh, Saudi Arabia; d Critical Care Unit, University Hospital of Birmingham NHS Foundation Trust, Birmingham, UK

**Keywords:** Acute kidney injury, COVID-19, acute respiratory distress syndrome, kidney replacement therapy, mortality, systematic review, meta-analysis

## Abstract

**Background:**

Acute kidney injury (AKI) is common among patients with COVID-19. However, AKI incidence may increase when COVID-19 patients develop acute respiratory distress syndrome (ARDS). Thus, this systematic review and meta-analysis aimed to assess the incidence and risk factors of AKI, need for kidney replacement therapy (KRT), and mortality rate among COVID-19 patients with and without ARDS from the first wave of COVID-19.

**Methods:**

The databases MEDLINE and EMBASE were searched using relevant keywords. Only articles available in English published between December 1, 2019, and November 1, 2020, were included. Studies that included AKI in COVID-19 patients with or without ARDS were included. Meta-analyses were conducted using random-effects models.

**Results:**

Out of 618 studies identified and screened, 31 studies met the inclusion criteria. A total of 27,500 patients with confirmed COVID-19 were included. The overall incidence of AKI in patients with COVID-19 was 26% (95% CI 19% to 33%). The incidence of AKI was significantly higher among COVID-19 patients with ARDS than COVID-19 patients without ARDS (59% vs. 6%, *p* < 0.001). Comparing ARDS with non-ARDS COVID-19 cohorts, the need for KRT was also higher in ARDS cohorts (20% vs. 1%). The mortality among COVID-19 patients with AKI was significantly higher (Risk ratio = 4.46; 95% CI 3.31–6; *p* < 0.00001) than patients without AKI.

**Conclusion:**

This study shows that ARDS development in COVID-19-patients leads to a higher incidence of AKI and increased mortality rate. Therefore, healthcare providers should be aware of kidney dysfunction, especially among elderly patients with multiple comorbidities. Early kidney function assessment and treatments are vital in COVID-19 patients with ARDS.

## Background

In December 2019, a novel coronavirus disease (COVID-19), was identified in Wuhan, China, which has spread quickly worldwide [1]. On March 11, 2020, the World Health Organization declared COVID-19 as a pandemic [2]. At the time of writing this review, over 32.7 million COVID-19 cases and 991,000 deaths have been reported. This rapid spread of COVID-19 across the world brought many challenges to global health care systems. COVID-19 leads to a wide range of clinical manifestations from asymptomatic, mild, moderate to severe health conditions. Symptomatic patients usually present with fever, dry cough, shortness of breath, myalgia, or fatigue [3]. COVID-19 is of clustering onset and mostly affects the respiratory system, with some patients rapidly progressing to acute respiratory distress syndrome (ARDS); these patients frequently require admission to the intensive care unit (ICU) [4]. Furthermore, it has been demonstrated that severe cases are mostly among the elderly and those with an underlying disease, which may progress to respiratory failure and increased risk of mortality [4].

A growing body of literature has exhibited that there are three potential mechanisms through which the kidney becomes involved in COVID-19 disease: cytokine-induced damage, systemic effects, and organ crosstalk [5]. These mechanisms are interrelated and have important implications for extracorporeal therapy [5]. Several risk factors may contribute to developing acute kidney injury (AKI) in severe cases; for example, most COVID-19 patients with AKI are older. Furthermore, they have comorbidities such as diabetes mellitus or hypertension, which are well-known to associate with kidney vulnerability [6]. ARDS has been identified as one of the prominent conditions that could lead to AKI development; this is also one of the primary reasons for admission in individuals with severe COVID-19 [4,7,8]. Additionally, some factors were associated with AKI development in patients with ARDS, such as impairment of gas exchange and severe hypoxemia [9].

Early reports from China suggested that the prevalence of AKI among patients with COVID-19 was low [6]. Guan et al. studied 1,099 Chinese patients with confirmed COVID-19; 93.6% of those patients were hospitalized, but only 5.3% of those were admitted to the ICU [7]. Across the hospital cohort, 3.4% developed ARDS and only 0.5% developed AKI. Notwithstanding, AKI is mostly common in critically ill patients with COVID-19, affecting 20–40% of patients admitted to ICU [10]. It is deemed a marker of disease severity and an adverse prognostic factor of survival [10]. A multicentre retrospective study from Wuhan investigated 239 critically ill COVID-19 patients and reported that the most common complications among those patients were ARDS, acute cardiac injury, and AKI (68.6%, 43.1%, and 49.8%, respectively) [1].

To date, most studies have revealed the incidence of AKI and ARDS in COVID-19 patients. However, none of these studies has compared AKI incidence in COVID-19 patients with and without ARDS. Therefore, this systematic review aimed to assess the incidence and risk factors of AKI among COVID-19 patients with and without ARDS. We also aimed to assess the necessity of kidney replacement therapy (KRT) in addition to the mortality rate.

## Materials and methods

The protocol of this review has been registered prospectively in PROSPERO (CRD42020219460). The Preferred Reporting Items for Systematic Reviews and Meta-Analyses statement (PRISMA) guideline was used to report this systematic review and meta-analysis [11]. The PRISMA checklist is provided in Supplementary 1.

A comprehensive literature search was performed by searching MEDLINE and EMBASE databases *via* the Ovid portal to select studies that met the inclusion criteria. The search terms and strategy used were; (Adult*) AND (“Novel coronavirus.ti,ab.” OR “New coronavirus.ti,ab.” OR “Coronavirus 2019.ti,ab.” OR “COVID-19.ti,ab.” OR “SARS-CoV-2.ti,ab.”) AND (“hospitalisation.ti,ab.” OR “hospitalization.ti,ab.” OR “oxygen therapy.ti,ab.” OR “ventilation.ti,ab.” OR “mechanical ventilation.ti,ab.” OR “Positive Pressure Ventilation.ti,ab.” OR “renal dialysis.ti,ab.” OR “renal replacement therapy.ti,ab.” OR “RRT.ti,ab”). AND (“acute kidney injury.ti,ab.” OR “AKI.ti,ab.” OR “acute renal injury.ti,ab.” OR “acute kidney insufficiency.ti,ab.” OR “acute renal insufficiency.ti,ab.” OR “acute kidney failure.ti,ab.” OR “acute renal failure.ti,ab.” OR “respiratory distress syndrome.ti,ab.” OR “respiratory failure.ti,ab.”) (Supplementary 2). The search was limited to original research published between December 1, 2019 and November 1, 2020 with full text available in English.

### Study selection

Two authors independently screened the abstracts, and any disagreements were resolved by consensus or by a third reviewer. The studies were initially screened by title and abstract to assess articles for relevance. The following populations were excluded: pediatric, end-stage kidney disease, kidney transplant, pregnant, or patients who have cancer. Additionally, case reports, reviews, and case series with less than ten patients were excluded. The full texts for the remaining studies were assessed for the following inclusion criteria: observational cohort studies, cross-sectional studies, and case series, with extractable quantitative data on patient demographics and data on AKI, ARDS, need for KRT, and mortality rate. Retrieved full-texts were independently assessed for eligibility by two authors, and disagreements were resolved through consensus.

### Data extraction and quality assessment

The following information was extracted from each included study: authors’ names, study sites, study design, sample size, cohort demographics (age and sex), the prevalence of comorbidities, and ICU admission. All clinical information related to AKI incidence, ARDS, need for KRT, and the mortality rate was extracted. The primary outcome was the incidence of AKI in COVID-19 patients with and without ARDS. The secondary outcomes were the need for KRT and the mortality rate of COVID-19 patients with AKI and ARDS as well as AKI without ARDS. All studies were qualitatively evaluated based on the National Institute of Health (NIH) quality assessment tools for observational cohort/cross-sectional studies and case series studies with the quality classified as good, fair, and poor. Two independent reviewers assessed the quality of included studies (FA and MAA). Disagreement was resolved through discussion.

### Data synthesis and analysis

Random-effects meta-analyses were performed to obtain pooled incidence, Risk Ratio (RR) estimates, and 95% confidence intervals (CIs) for categorical variables. The Metan and Metaprop command tools were used to analyze the incidence of AKI [12]. After the initial analysis, heterogeneity in incidence and mortality outcomes were investigated. All analyses were performed using Stata 16 and Revman 5 (Cochrane). Funnel plot were used to assess publication bias.

## Results

### Study selection

**Figure 1.**
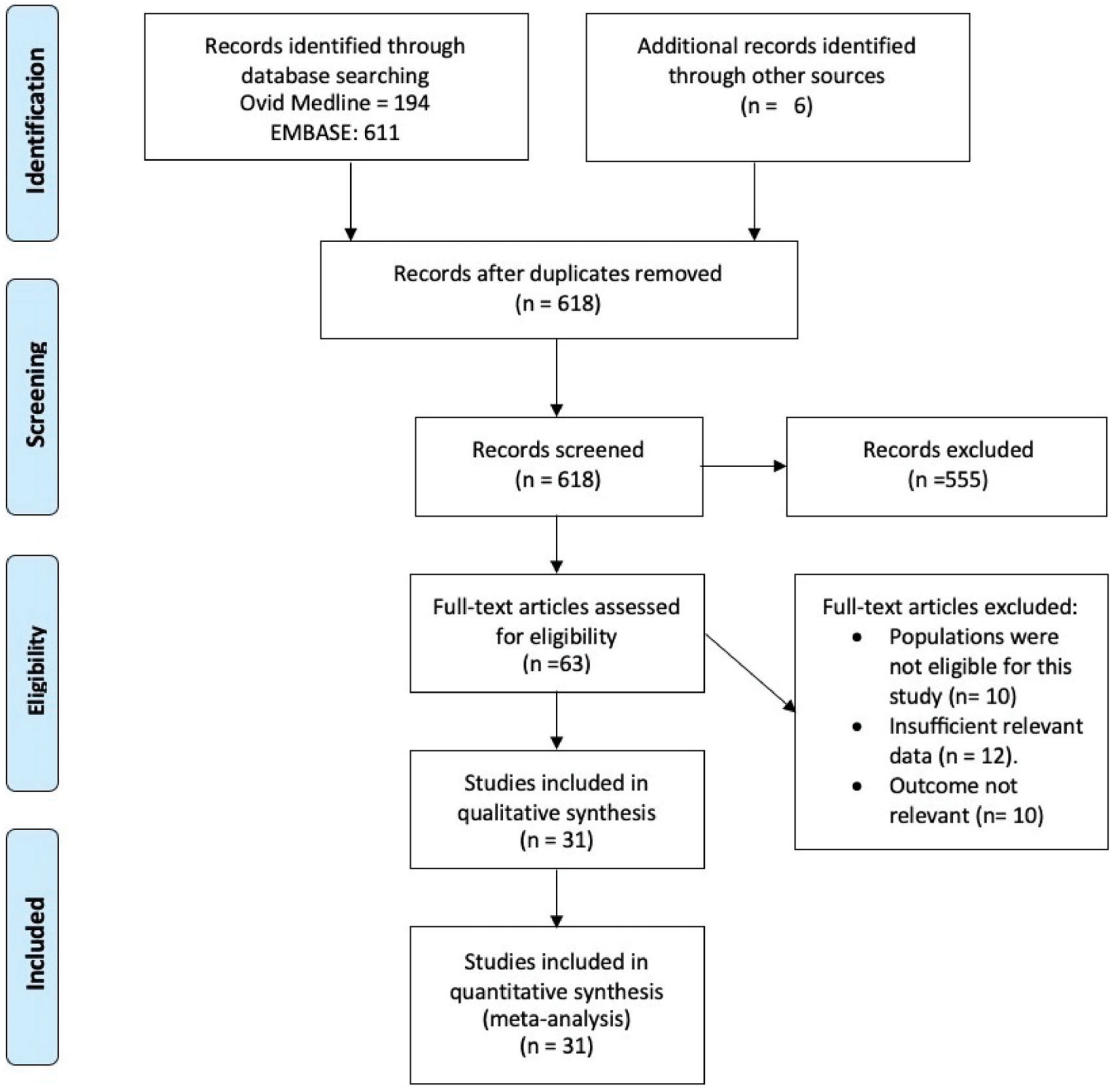
PRISMA flow diagram.

A total of 618 abstracts were obtained and screened, of which 63 full-text articles were assessed for eligibility and 31 met the inclusion criteria (Figure 1 shows the PRISMA flow diagram). Only two studies were cross-sectional in design [13,14], fifteen studies were cohort studies [15–29], and fourteen studies were case series [7,8,30–41].

### Study characteristics

The included studies covered 27,500 patients from hospitals in China, United States (US), United Kingdom (UK), Morocco, Italy, France, and Belgium. All studies included hospitalized patients, except Guan et al. [7], who included both inpatients and outpatients. Seven studies included only patients admitted to the ICU. All included studies reported the incidence of AKI and mortality rate; twenty-four studies reported the need for KRT; twenty-one studies reported the development of ARDS in COVID-19 patients.

The majority of patients were male (55.9%, 15378/27500). Twenty-one per cent (5934/27500) were admitted to the ICU, and 16.8% of all patients (4617/27128) died. The most common comorbidities reported were hypertension (40%) and diabetes (26%). 5.8% of patients had evidence of chronic kidney disease (CKD) at baseline. Two studies from United States [30,37] and one from Italy [27] reported CKD prevalence between 28.6% to 47.6% (See Table 1).

**Table 1.** Characteristics of the included studies in patients with COVID-19.

				Demographics		Comorbidities (%)			
Study name	Country	Study design	*N*	Mean/median age (years)	Male (%)	Hypertension	Diabetes	CKD	Obesity
Aggarwal et al. [15]	US	Single-centre, retrospective cohort study	16	67 (38–95)	12 (75)	9 (57)	5 (31)	6 (38)	8 (50)
Arentz et al. [30]	US	Single-centre, retrospective case series	21	70 (43–92)	11 (52)	__	7 (33.3)	10 (47.6)	__
Argenziano et al. [31]	US	Single-centre, retrospective case series	1000	63 (50–75)	596 (59.6)	601 (60.1)	372 (37.2)	137 (13.7)	52/841 (48.3)
Ch et al. [32]	US	Multicentre, Retrospective case series	300	58.2 ± 12.6	182 (60.7)	200 (66.7)	134 (44.7)	39 (13)	163 (54)
Chen, Bai et al. [16]	China	Multicentre, Retrospective Cohort study	3309	62 (49–69)	1642 (49.62)	988 (29.9)	464 (14)	57 (1.7)	__
Chen, Liu et al. [17]	China	Multicentre, Retrospective Cohort study	792	55 (36‐68)	432 (55)	215 (27)	142 (18)	26 (3.3)	__
Cheng et al. [18]	China	Single-centre, retrospective cohort study	1392	63 (50–71)	711 (51)	499 (36)	241 (17)	21 (1.5)	__
Cheng et al. [33]	China	Single-centre, Prospective case series	701	63 (50–71)	367 (52.4)	233 (33.4)	100 (14.3)	14 (2)	__
Cui et al. [19]	China	Multicentre, Retrospective cohort study	116	59	66 (56.9)	38 (32.27)	28 (24.1)	5 (4.3)	__
El Aidaoui et al. [20]	Morocco	Single-centre, retrospective cohort study	134	53 (36–64)	73 (54.5)	36 (26.9)	19 (14.2)	3 (2.2)	__
Fisher et al. [21]	US	Multicentre, Retrospective cohort study	3345	64.4 ± 16.4	1776 (53.1)	__	906 (27.1)	409 (12.1)	1351 (42.9)
Fominskiy et al. [22]	Italy	Single-centre, Prospective cohort study	99	61	83 (83.8)	34 (52.9)	12 (13.6)	5 (5)	22 (22.2)
Gasparini et al. [14]	UK	Multicentre, Retrospective cross-sectional study	372	59 (51–65)	269 (72)	__	__	__	__
Grimaldi et al. [23]	France/Belgium	Multicentre, Retrospective cohort study	415	64 ± 10	321 (77)	235 (57)	__	33 (7.9)	__
Guan et al. [7]	China	Multicentre, Retrospective case series	1099	47 (35–58)	640 (58.2)	165 (15)	81 (7.4)	8 (0.7)	__
Hirsch et al. [34]	US	Multicentre, Retrospective case series	5449	64 (52 − 75)	3317 (60.9)	3037(55.7)	1797 (33)	__	1475 (27.1)
Hong et al. [24]	China	Single-centre, retrospective cohort study	98	55.4 ± 17.1	38 (38.8)	30 (30.6)	9 (9.2)	__	__
Huang et al. [8]	China	Single-centre, Prospective case series	41	49 (41–58)	30 (73.2)	6 (15)	8 (20)	__	__
Okoh et al. [25]	US	Single-centre, retrospective cohort study	251	62 (49–74)	129 (51)	175 (70)	115 (46)	46 (18)	120 (48)
Pei et al. [35]	China	Single-centre, retrospective case series	333	56.3 ± 13.4	182 (54.7)	107 (32.2)	76 (22.9)	__	__
Qian et al. [26]	China	Single-centre, retrospective cohort study	37	55 (48–68)	26 (68.4)	14 (36.8)	8 (21.1)	1 (2.7)	__
Richardson et al. [36]	US	Multicentre, Retrospective case series	5700	63 (52–75)	3437 (60.3)	3026 (56.6)	1808 (33.8)	268 (4.7)	1737 (41.7)
Russo et al. [27]	Italy	Single-centre, retrospective cohort study	777	70 ± 16	59 (7.6)	385 (49.5)	123 (15.8)	222 (28.6)	__
Suleyman et al. [37]	US	Single-centre, retrospective case series	463	57.5 ± 16.8	204 (44)	295 (63.7)	178 (38.4)	182 (39.3)	262 (57.6)
Yang et al. [38]	China	Single-centre, retrospective case series	52	59.7 ± 13.3	35 (67)	__	9 (17)	__	__
Yu et al. [13]	China	Multicentre, Prospective cross-sectional study	226	64 (57–70)	139 (61.5)	96 (42.5)	47 (20.8)	__	__
Zahid et al. [28]	US	Single-centre, retrospective cohort study	469	66 (55 − 75)	268 (57.1)	232 (68.6)	219 (46.7)	50 (11)	__
Zangrillo et al. [39]	Italy	Single-centre, retrospective case series	73	61 (54–69)	61 (83.6)	39 (52.9)	10 (13.6)	__	__
Zheng et al. [40]	China	Single-centre, retrospective case series	34	66 (58 − 76)	23 (67.6)	22 (64.7)	8 (23.5)	2 (5.9)	__
Zhou, Yang et al. [41]	China	Multicentre, Retrospective case series	195	66 (56–76)	130 (66.7)	89 (45.6)	55 (28.2)	__	__
Zhou, Yu et al. [29]	China	Multicentre, Retrospective Cohort study	191	56 (46–67)	119 (62)	58 (30)	36 (19)	2 (1)	__

Values are median (IQR), mean (SD) or number (proportion).

N: number; CKD: Chronic kidney disease.

Definitions of AKI and ARDS were consistent among the included studies, and the same definitions were used in this review. AKI was defined based on the Kidney Disease: Improving Global Outcomes criteria (KDIGO) [42] and ARDS was defined based on Berlin definition [43].

### Quality assessment and risk of bias

**Figure 2.**
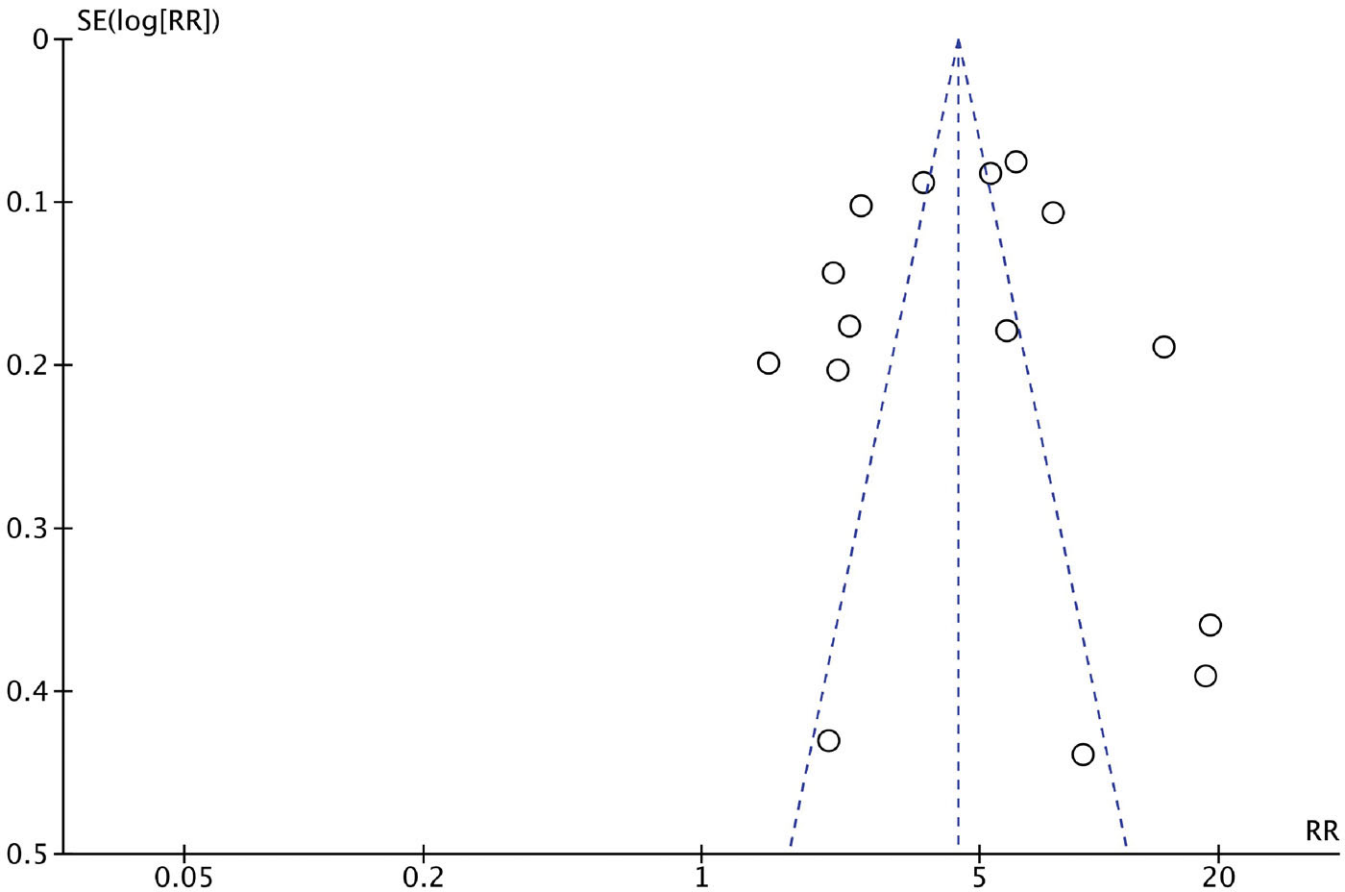
Funnel plot for detection of publication bias.

As the studies were case-series and cohort/cross-sectional studies, all included articles were evaluated using the NIH quality assessment tools. Domains 5 of the NIH quality assessment tool of case series and also the domain 5, 8, and 12 of the NIH quality assessment tool of observational cohort/cross-sectional studies were not applicable as they relate to the intervention, power calculation, exposure measure overtime and blinding of outcome assessment and these were not applicable to the included studies (Supplementary 3). Out of the 31 studies, 27 were judged as good quality, 3 as fair [15,19,28] and 1 as poor quality [24]. A funnel plot did not indicate major publication bias (Figure 2).

### Results of individual studies

Across all 31 studies, a total of 27,500 patients were included in this review. Overall pooled AKI incidence was 26% (95% CI 19–33). Across eight studies that only included COVID-19 patients admitted to the ICU (*n* = 1540), AKI incidence was 50% (95% CI 35–65) [13,14,22,23,30,32,39,40]. Only three studies included hospitalized patients who were not admitted to the ICU, across which the pooled AKI incidence was only 25% (95% CI 21–29) [27,28,38]. In Table 2, the overall study outcomes are reported.

**Table 2.** Outcomes of the included studies in patients with COVID-19.

				Incidence			Mortality			
Study name	Country	*N*	Admitted to ICU	AKI	ARDS	Need for KRT	Overall mortality	AKI	ARDS	KRT
Aggarwal et al. [15]	US	16	8 (50)	11 (69)	5 (31)	–	3 (19)	–	–	–
Arentz et al. [30]	US	21	–	4 (19.1)	20 (95.2)	–	11 (52.4)	–	–	–
Argenziano et al. [31]	US	1000	236 (23.6)	288/850 (33.9)	299/850 (35.2)	117/850 (13.8)	211 (21.1)	–	–	–
Ch et al. [32]	US	300	300 (100)	230 (76.7)	275 (91.7)	133 (44.3)	157(52.3)	138 (60)	155 (56.4)	82 (61.7)
Chen, Bai et al. [16]	China	3309	1036 (31.3)	401 (12.1)	1325 (40)	–	307 (9.3)	–	–	–
Chen, Liu et al. [17]	China	792	94 (12)	46 (6)	104 (13)	11 (1.4)	68 (8.6)	32 (69.6)	61 (58.6)	11 (100)
Cheng et al. [18]	China	1392	140 (10)	99 (7)	–	–	199 (14)	71 (71.7)	–	–
Cheng et al. [33]	China	701	73 (10.4)	36 (5.1)	–	–	113 (16.1)	–	–	–
Cui et al. [19]	China	116	NA	21 (18.1)	–	–	18 (15.5)	12 (57.1)	–	–
El Aidaoui et al. [20]	Morocco	134	45 (33.6)	8 (6)	13 (9.7)	8 (6)	14 (10.4)	8 (100)	4 (30.8)	–
Fisher et al. [21]	US	3345	438 (13.1)	1903 (56.9)	–	164 (4.9)	775 (23.2)	641 (33.7)	–	–
Fominskiy et al. [22]	Italy	99	99 (100)	72 (72.7)	–	17 (17.7)	33 (33.3)	28 (38.9)	–	9 (52.9)
Gasparini et al. [14]	UK	372	372 (100)	168 (45)	–	121 (32.5)	139 (37)	81 (48)	–	–
Grimaldi et al. [23]	France /Belgium	415	415 (100)	231 (56)	415 (100)	82 (19.76)	166 (40)	121 (52.4)	166 (40)	49 (59.7)
Guan et al. [7]	China	1099	33 (16.2)	6 (0.5)	37 (3.4)	9 (0.8)	15 (1.4)	–	–	–
Hirsch et al. [34]	US	5449	1395 (25.6)	1993 (36.6)	–	285 (5.2)	888 (16.3)	694 (34.8)	–	157 (55.1)
Hong et al. [24]	China	98	13 (13.3)	9 (9.2)	18 (18.4)	3 (3.1)	5 (5.1)	–	–	–
Huang et al. [8]	China	41	13 (32)	3 (7)	12 (29)	3 (7)	6 (14.6)	–	–	–
Okoh et al. [25]	US	251	82 (33)	52 (21)	82 (33)	52 (21)	97 (38.6)	–	–	–
Pei et al. [35]	China	333	189 (56.8)	35 (10.5)	–	6 (1.8)	29 (8.7)	20 (57.1)	–	–
Qian et al. [26]	China	37	37 (100)	8 (21.6)	11 (29.7)	3 (8.1)	1 (2.7)	–	–	–
Richardson et al. [36]	US	5700	373 (6.5)	1370 (24)	–	225 (3.9)	553 (9.7)	347 (25.3)	–	78 (34.7)
Russo et al. [27]	Italy	777	–	176 (22.6)	–	21 (2.8)	275 (35)	111 (63.1)	–	–
Suleyman et al. [37]	US	463	141 (39.7)	159/355 (44)	111/355 (31.3)	25/355 (7)	72 (16)	–	–	–
Yang et al. [38]	China	52	–	15 (29)	35 (67)	9 (17)	32 (61·5)	12 (80)	26 (74.3)	8 (88.9)
Yu et al. [13]	China	226	226 (100)	57 (25.2)	161 (71.2)	24 (10.6)	87 (38.5)	–	–	–
Zahid et al. [28]	US	469	–	128 (27.3)	206 (44)	22 (4.7)	188 (40.1)	91 (71.1)	–	18 (81.8)
Zangrillo et al. [39]	Italy	73	73 (100)	55 (75.3)	73 (100)	16 (21.9)	19 (26)	–	19 (26)	–
Zheng et al. [40]	China	34	34 (100)	7 (20.6)	33 (97.1)	5 (14.7)	0 (0)	–	–	–
Zhou, Yang et al. [41]	China	195	19 (9.8)	58 (29.7)	162 (83.1)	26 (13.3)	82 (42)	–	–	–
Zhou, Yu et al. [29]	China	191	50 (26)	28 (15)	59 (31)	10 (5)	54 (28.3)	27 (96)	50 (84.7)	10 (100)

Values are number (proportion).

N: number; ICU: Intensive care unit; AKI: Acute Kidney Injury; ARDS: Acute respiratory distress syndrome; KRT: Kidney Replacement Therapy.

The majority (68.6%) of patients admitted to ICU had ARDS [13,14,22,23,26,32,39,40]. Suleyman et al. [37] carried out a case series of 463 consecutive patients with COVID-19. The mean age was 57.5 ± 16.8 years and the majority were female (55.9%), and African American (72.1%). The most common comorbidities in this cohort included hypertension (63.7%), CKD (39.3%), and diabetes (38.4%). Of these patients, 355 required hospital admission; 214 were treated in the general practice unit and 141 required ICU management. The AKI incidence was significantly higher among patients in the ICU than patients in the general practice unit (69.5% vs 28.5%. *p* < 0.001). In the UK, Gasparini et al. [14] conducted a multicentre observational study, aiming to examine the association between acute and chronic kidney disease with clinical outcomes in 372 patients with COVID-19 and admitted to ICU. The median age of this cohort was 59 (51–65) years. Most of them were male (72%) and the majority of patients were from a Black, Asian and minority ethnic (BAME) background (76%). Most patients (91%) required mechanical ventilation. A total of 168 patients (45%) developed AKI and had a high mortality rate (48%) (95% CI 41–56).

### AKI incidence rate in non-ARDS COVID-19 patients

After excluding all studies that reported the presence of ARDS among their cohorts, the pooled AKI incidence across eight studies with a total of 18,185 patients, was 23% (95% CI 13–35) [14,18,19,21,27,33–36]. There was a high rate of comorbidities in these cohorts including hypertension (42%) (95% CI 35–49) [18,19,27,33–36], obesity (33%) (95% CI 26–40) [21,34,36], and diabetes (23%) (95% CI 18–28) [18,19,21,27,33–35].

Hirsch et al. [34] and Richardson et al. [36] studied 11,149 hospitalized COVID-19 patients with a mean age of 63.5 (52–75) years. Both cohorts had a high prevalence of hypertension (55.7%–56.6%) and obesity (27.1%–41.7%). Both studies reported a high incidence of AKI (24%–36.6%) and a high mortality rate in those patients who developed AKI (25.3%–35%).

Furthermore, Cheng et al. [18] studied 1392 COVID-19 patients retrospectively, aiming to assess AKI incidence. The median age of this cohort was 63 years, and they had fewer comorbidities compared to previous studies: hypertension (36%), diabetes (17%) and CKD (1.5%). Only 7% (99 patients) of this cohort developed AKI, and no patient needed KRT. Later, the same authors conducted a prospective cohort study on 701 COVID-19 patients [33]. The median age was 63 years, and 33.4% of those patients had hypertension, 17% had diabetes, and only 2% had CKD. The incidence of AKI among those patients was 5.1%, and the overall mortality was 16.1%.

### AKI incidence rate in ARDS COVID-19 patients

Twenty-two studies included COVID-19 patients with ARDS. While these cohorts included >90% of patients with ARDS, the pooled AKI incidence was 67% (95% CI 57–76) [22,23,28,32,39,40]. Grimaldi et al. [23] examined 415 COVID-19 patients with moderate-to-severe ARDS who were invasively ventilated and treated with different antiviral strategies. Patients were aged between 50 and 70 years. Most were males (*n* = 321, 77%) with mean body mass index (BMI) (30 ± 5 kg/m2), and hypertension was the most frequent comorbidity among the cohort (*n* = 235 patients; 57%). The findings of this study showed that 56% of patients developed AKI. However, these findings might be affected by the different antiviral therapies being used. Zangrillo et al. [39] also assessed 73 COVID-19 patients with ARDS; they were mostly male (83.6%) with a median age of 61 years. The authors revealed that 75.3% of the population developed AKI. The most frequent comorbidities were hypertension (52.9%), obesity (>25%) and diabetes (13.6%). Fominskiy et al. [22] studied 99 confirmed COVID-19 patients who were admitted to ICU and invasively ventilated. The aim was to assess the clinical characteristics, predictors of AKI, and KRT need. The median age was 61 years, and the most frequent comorbidities were hypertension (52.9%) and obesity (22.2%). Since all patients were invasively ventilated, it is likely that a substantial proportion of these patients had ARDS. A total of 72.7% of those patients developed AKI.

Ch et al. [32] studied 300 COVID-19 patients admitted to the ICU with a mean age (+SD) of 58.2 ± 12.6 years. The majority (91.7%) of participants had ARDS, and 76.7% of patients presented either with AKI on admission or developed AKI during the course of illness. Increasing age (RR: 1.02; 95% CI 1.01–1.03)) and male sex (48.4% vs. 23.8%; RR: 1.68; 95% CI 1.2–2.3) were also risk factors for presentation with AKI. Both Arentz et al. [30] and Zheng et al. [40] carried out similar case series that included 55 participants to assess the clinical characteristics and outcomes of confirmed COVID-19 patients admitted to the ICU. The mean age was between 66 to 70 years. Although the majority (96.36%) of participants in both studies had ARDS, AKI incidence was ranged from 19.1% to 20.6%. The most common comorbidities reported by Arentz et al. [30] were CKD (47.6%) and congestive heart failure (42.9%), and the most frequent comorbidity reported by Zheng et al. [40] was hypertension (64.7%).

### Need for KRT

Twenty-five studies reported the need for KRT and only 6% of all patients required KRT (Table 2). After analyzing the studies that reported patients with ARDS, we found that 10% of patients required KRT across nineteen studies. Nevertheless, the need for KRT was only 5% among studies that did not report ARDS incidence [14,21,27,34–36]. A higher incidence of AKI (76.7%) and need for KRT (44.3%) were reported by Ch et al. [32] who only included patients who were admitted to the ICU, of which >90% developed ARDS.

### Mortality

The overall mortality across all cohorts was 16.9%. However, the mortality was higher (38.7%) when the studies enrolled only COVID-19 patients admitted to the ICU [13,14,22,23,26,32,39,40]. The mortality rate was significantly higher among cohorts comprising >90% of ARDS patients. For instance, ARDS patients comprised >90% of cohorts studied by both Ch et al. [32] and Arentz et al. [30], and the mortality was 52.3 and 52.4%, respectively. AKI mortality was reported across eighteen studies. Across eight studies that included patients with ARDS, AKI mortality was 60.3% (range, 52.4%–100%) [13,17,20,22,23,29,32,39]. The remaining eight studies did not report ARDS incidence and revealed that AKI mortality was 34.2% (range, 25.3%–63%) [14,18,19,21,27,34–36].

### Synthesis of results

**Figure 3.**
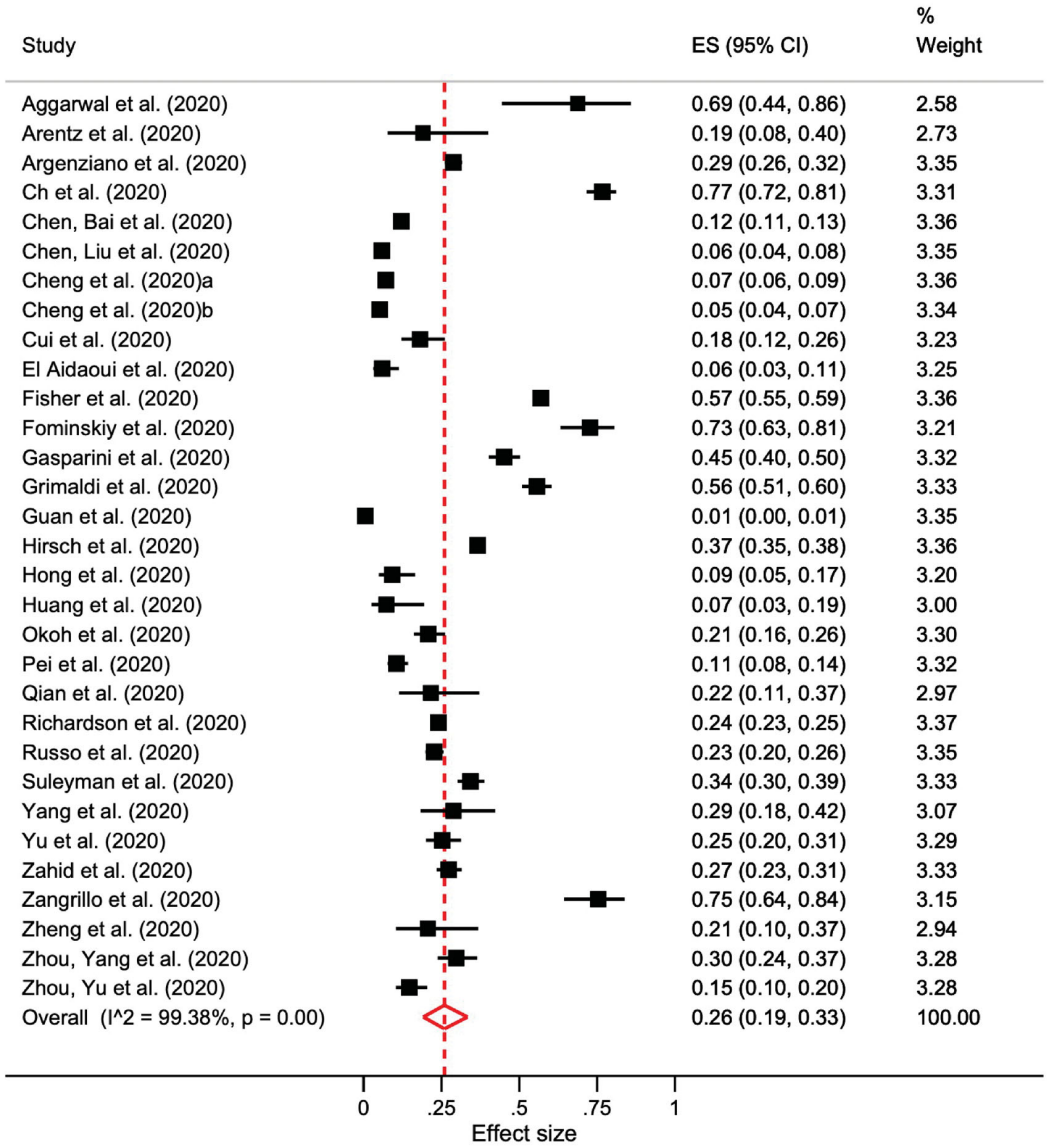
Forest plot of pooled AKI incidence in overall cohorts.

A total of 27,500 patients from 31 studies were included in the meta-analysis of AKI incidence amongst all COVID-19 cohorts. By using a random-effects model, the pooled incidence of AKI was 26% (95% CI 19–33; I^2^= 99.38%; *p* < 0.001; Figure 3).

**Figure 4.**
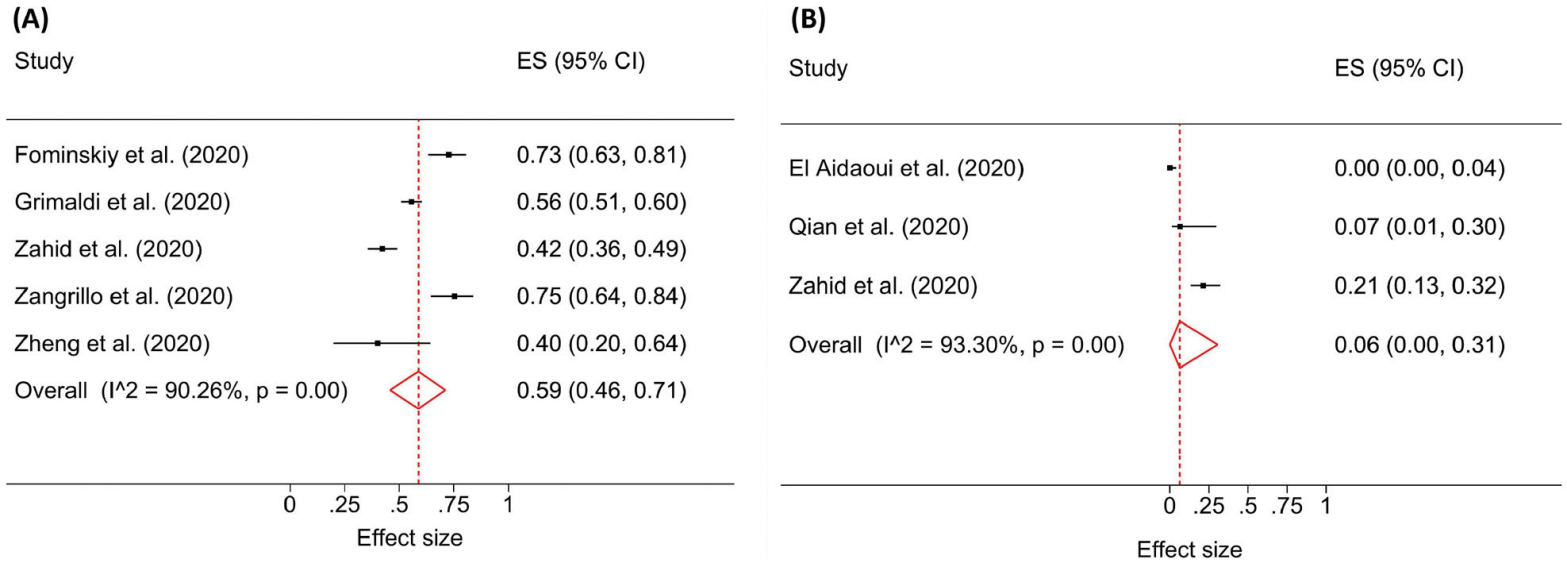
Forest plot of the pooled AKI incidence showing the increased rate in ARDS (A) compared to non-ARDS (B) cohorts.

A total of 808 COVID-19-ARDS patients from five studies were included. The pooled incidence of AKI in ARDS patients was 59% (95% CI 46–71; I^2^= 90.26%; *p* < 0.001; Figure 4A). A total of 174 COVID-19 patients without ARDS were included from three studies. The pooled incidence of AKI in this population was 6% (95% CI 0–31; I^2^ = 93.30%; *p* < 0.001; Figure 4B).

**Figure 5.**
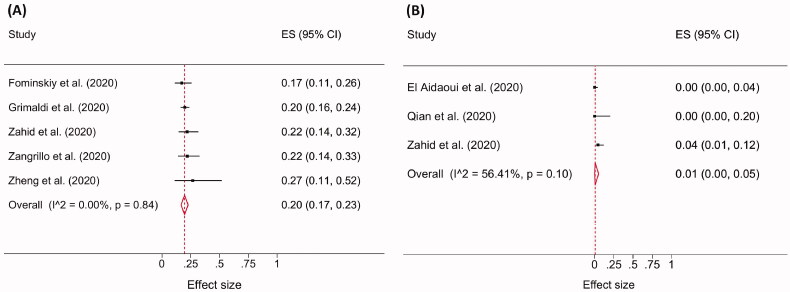
Forest plot demonstrating the need for KRT in ARDS (A) compared with non-ARDS (B) COVID-19 cohort.

The pooled need for KRT was assessed on a total of 590 ARDS patients from four studies and 174 non-ARDS patients from three studies. In ARDS patients, the pooled need for KRT was 20% (95% CI 17–23; I^2^ = 0; *p* = 0.84; Figure 5A) and only 1% (95% CI 0–5; I^2^ = 56.41%; *p* = 0.10; Figure 5B) in non-ARDS patients.

**Figure 6.**
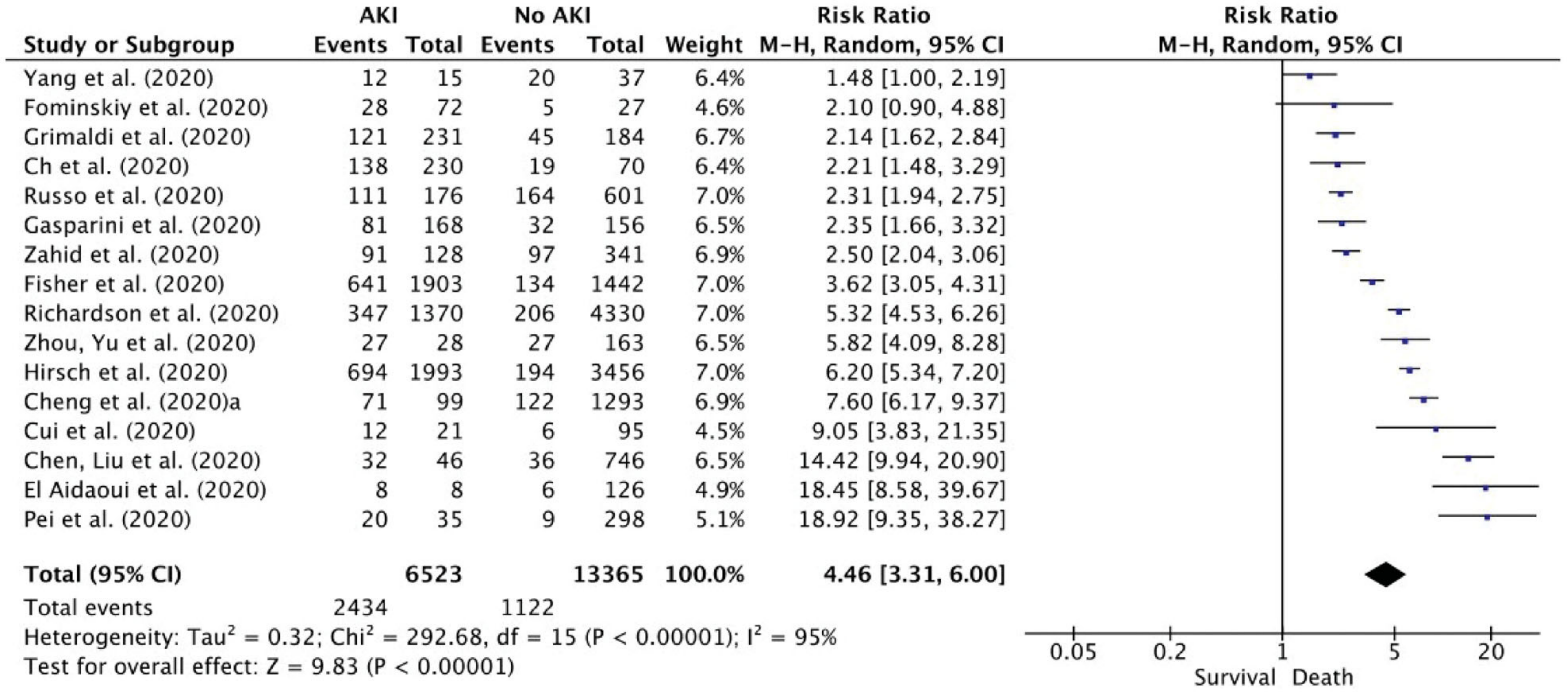
Forest plot for mortality risk among patients with AKI associated with COVID-19.

The AKI mortality was analyzed based on the data of 19,888 patients available from 16 studies. Development of AKI was associated with significantly higher mortality among COVID-19 patients, with RR of 4.46 (95% CI 3.31–6; I^2^= 95%; *p* < 0.00001; Figure 6) in a random-effects model.

## Discussion

To the authors' knowledge, this is the first systematic review to assess and compare the incidence of AKI between COVID-19 patients with and without ARDS. Based on the literature, the incidence of AKI in ARDS patients prior to the emergence of COVID-19 was approximately 35–50%, and was associated with a high mortality rate which significantly increased by almost two-fold in the ICU [44,45]. For instance, a retrospective study examined a cohort of 634 ARDS patients and revealed an AKI incidence rate of 68.3% after ARDS onset [46]. However, AKI incidence may increase significantly in COVID-19 patients after the onset of ARDS, suggesting lung-kidney crosstalk as the dominant cause of kidney injury [47].

This review shows that most of the included studies reported a higher incidence of AKI in COVID-19 patients who developed ARDS. Across all cohorts with confirmed COVID-19 infection from China, US, UK, Morocco, Italy, France, and Belgium, we identified a wide range of AKI incidence which ranged from 0.5% to 76.7%. However, this may reflect the variety of disease severity thresholds for hospitalization all over the world and potential differences in clinical practice for monitoring kidney dysfunction.

Our findings show that COVID-19 patients admitted to the ICU had a higher incidence of AKI; however, most (68.6%) of those patients also had ARDS. This finding is consistent with that of Xu et al. [1] who studied 239 critically ill COVID-19 patients admitted to the ICU and found that 49.8% of those patients developed AKI, and 68.6% had ARDS.

We identified that a high AKI incidence was most commonly reported when >90% of patients in the cohort had ARDS [22,23,30,32,39–41]. Additionally, this meta-analysis comparing ARDS with non-ARDS COVID-19 cohorts revealed that AKI incidence was significantly higher among COVID-19 patients with ARDS (59% vs 6%, *p* < 0.001). These findings are consistent with a multicentre study conducted in nine ICUs located in Europe, which found that 50% of COVID-19 patients with ARDS developed AKI [48].

Although some studies did not enroll COVID-19 patients with ARDS, a few studies demonstrated a high incidence of AKI that ranged from 5.1% to 56.9% [14,18,21,27,33–36]. Based on the literature, patients with a history of CKD are more likely to have comorbidities that have been identified as risk factors for poor outcomes, such as older age, hypertension, diabetes mellitus, cardiovascular disease, and this may be explained by our findings [49]. Additionally, Nadim et al. suggested that the advanced age, CKD, and high prevalence of other comorbidities (e.g., hypertension, diabetes, obesity, and heart failure) are all linked to worse outcomes and are risk factors for the development of AKI in patients with COVID-19 [50].

The metabolic syndrome is a cluster of risk factors (diabetes mellitus, hypertension, obesity) for developing cardiovascular disease and kidney abnormalities [51]. The consequences of the metabolic syndrome on kidney functions in COVID-19 patients are poorly descried Lohia et al. [52] investigated the association between metabolic syndrome and severe disease outcomes among 1871 patients with confirmed COVID‐19 diagnosis. Author found that patients with metabolic syndrome had significantly increased in the ICU admission (odds ratio [OR], 1.68; 95% CI, 1.36‐2.08; *p* < .001), and need for mechanical ventilation (OR, 1.90; 95% CI, 1.52‐2.37; *p* < .001), and mortality rate (OR, 1.40; 95% CI, 1.11‐1.75; *p* = .004). Patients with CKD were higher in the metabolic syndrome cohort compared to the none-metabolic syndrome cohort (14.7% vs. 9%; *p* < .001). Metabolic syndrome cohort may be at higher risk of developing AKI if they contract COVID-19, and this risk may be by background CKD. Chen et al. [53] recruited >6000 participants to examine the association between the metabolic syndrome and kidney impairment, and they found that the multivariate-adjusted risk for both CKD (defined as a GFR of <60 mL/min per 1.73 m2) and microalbuminuria was significantly higher in those with the metabolic syndrome compared to participants without metabolic syndrome (Odds ratio 2.60, 95% CI 1.68–4.03 and 1.89, CI 1.34–2.67, respectively). Also, they revealed that risk increased progressively with the number of the metabolic syndrome’s components observed in each patient.

A meta-analysis comparing ARDS with non-ARDS COVID-19 cohorts showed the need for KRT was also higher in ARDS cohorts (20% vs 1%). Across all studies which reported the need for KRT, we observed a higher need for KRT among patients with a high incidence of AKI as reported by Ch et al. [32] and Fominskiy et al. [22]. However, it is unclear from these studies whether KRT was used to treat fluid overload, end-stage kidney disease, or patients with AKI alone.

The mortality rate was significantly increased in critically ill patients if they had a high incidence of AKI or ARDS [23,32]. In our meta-analysis, the overall mortality among patients with AKI was significantly higher than those without AKI (*p* < 0.00001). In both Ch et al. [32] and Arentz et al. [30], AKI and ARDS development was associated with a high mortality rate. Ch et al. [32] found that the AKI incidence was associated with a 25% increased risk of death (RR: 1.25; 95% CI: (1.02, 1.52)). These findings are consistent with previously published research, which found that severe AKI in patients with COVID-19 is associated with a high mortality rate [54]. Additionally, in the population of Ch et al. [32] high rates of comorbidities such as obesity (85.7% with BMI >25) and hypertension (66.7%) were also linked with increased mortality rate. Both Gasparini et al. [14] and Fominskiy et al. [22] also found that patients with AKI had a higher mortality rate compared to patients without AKI. A possible explanation for the higher mortality rate in the previous two studies is that each study recruited >90% critically ill individuals who were invasively ventilated in ICU.

## Limitations

The main limitation of this study is that most of the selected studies were of the retrospective design, making them susceptible to selection bias. There was limited data on AKI patients' characteristics among included studies, such as urine output status, the need for KRT and the grade of AKI. This information is essential to define the severity and recoverability of AKI. Moreover, most cohorts were from China and the United States, and a small amount from Africa and Europe, which does reduce the generalization to other countries. The heterogeneity between studies was high; consequently, most of the analyses were interpreted based on a random-effects model. Furthermore, given the rapid, constant expansion of COVID-19 research, several studies had limitations in their description of details and relatively short periods of follow-up. Finally, we cannot be certain that all relevant studies were captured, despite a detailed, comprehensive search strategy.

## Conclusion

A higher incidence of AKI was most common among COVID-19 cohorts admitted to the ICU and was associated with the development of ARDS. Also, increased age, male sex, and preexisting comorbidities (e.g., hypertension, diabetes, obesity, and CKD) were associated with significantly worse outcomes, which may be considered risk factors for developing AKI and increased mortality among COVID-19 patients. Therefore, healthcare providers should be aware of kidney dysfunction among those patients, especially those who are elderly with multiple comorbidities. This may ultimately lead to using appropriate diagnostic tools and treatment strategies to avoid additional damage to the kidneys and providing adequate kidney support when needed.

## Supplementary Material

Supplementary Material 3

Supplementary Material 2

Supplementary Material 1

## Data Availability

All data relevant to the study are included in the article or uploaded as supplementary information.

## Abbreviations

ARDS: Acute respiratory distress syndrome
BAME: Black, Asian and minority ethnic
CKD: Chronic kidney disease
COVID-19: Coronavirus disease 2019
ICU: Intensive care unit
KDIGO: Kidney Disease: Improving Global Outcomes
PRISMA: Preferred Reporting Items for Systematic Reviews and Meta-Analyses
RR: Risk ratio
KRT: Kidney replacement therapy
BMI: Body mass index
NIH: National Institute of Health.
