# Supplementary material for: Incidence and risk factors of acute kidney injury in COVID-19 patients with and without acute respiratory distress syndrome (ARDS) during the first wave of COVID-19: a systematic review and Meta-Analysis
Source: Ren Fail. 2021 Dec 9;43(1):1621–33. doi: 10.1080/0886022X.2021.2011747 (PMC8667924; doi:10.1080/0886022X.2021.2011747)
Supplement: Supplementary Material 3 [file IRNF_A_2011747_SM0505.pdf]

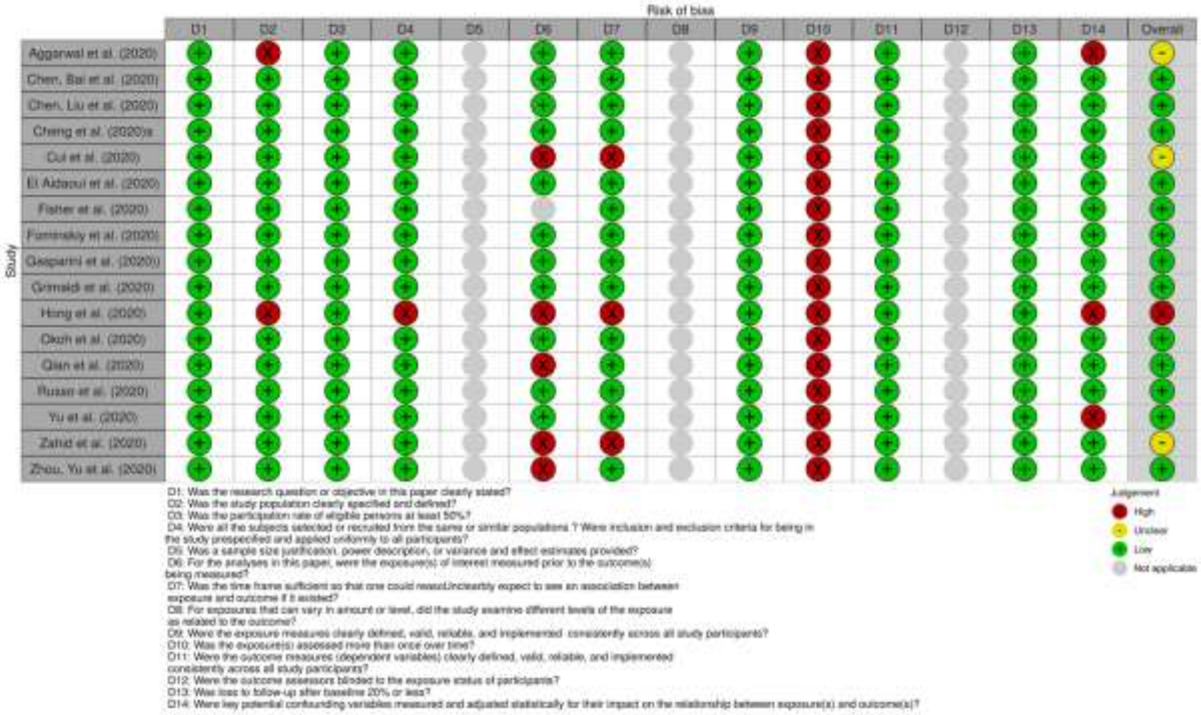

Figure 1: Quality assessment of included studies based on the NHLBI Observational Cohort/ Cross-Sectional Studies Quality Assessment Tool.

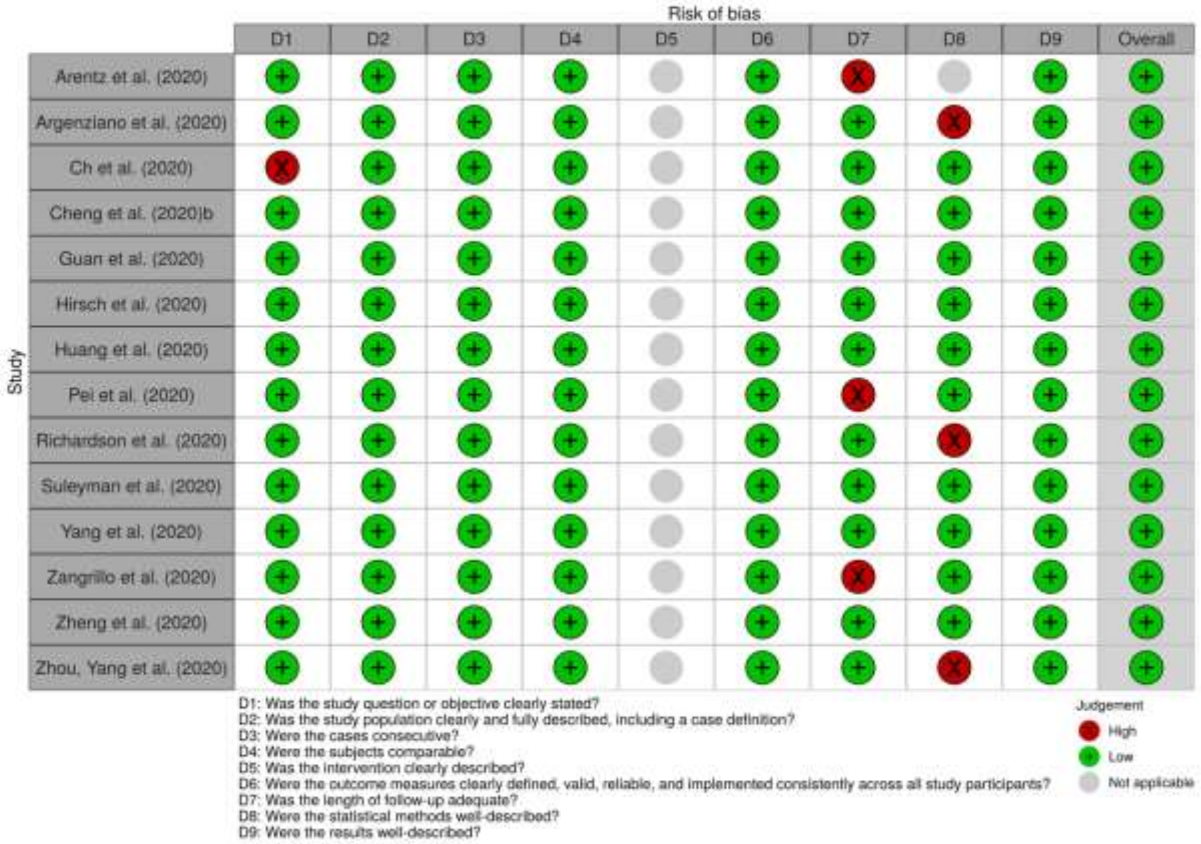

Figure 2: Quality assessment of included studies based on the NHLBI Case Series Quality Assessment Tool.
