# Supplementary material for: Incidence and risk factors of acute kidney injury in COVID-19 patients with and without acute respiratory distress syndrome (ARDS) during the first wave of COVID-19: a systematic review and Meta-Analysis
Source: Ren Fail. 2021 Dec 9;43(1):1621–33. doi: 10.1080/0886022X.2021.2011747 (PMC8667924; doi:10.1080/0886022X.2021.2011747)
Supplement: Supplementary Material 2 [file IRNF_A_2011747_SM0492.pdf]

Please find below the search terms were used via MEDLINE and EMBASE:

1. Adult*.mp.
2. Novel coronavirus.ti,ab.
3. New coronavirus.ti,ab.
4. Coronavirus 2019.ti,ab.
5. COVID-19.ti,ab.
6. SARS-CoV-2.ti,ab.
7. hospitalization.ti,ab.
8. hospitalisation.ti,ab.
9. oxygen therapy.ti,ab.
10. ventilation.ti,ab.
11. Mechanical ventilation.ti,ab.
12. Positive Pressure Ventilation.ti,ab.
13. Renal Dialysis.ti,ab.
14. Renal replacement therapy.ti,ab.
15. RRT.ti,ab.
16. Acute Kidney Injury.ti,ab.
17. AKI.ti,ab.
18. Acute Renal Injury.ti,ab.
19. Acute Kidney Insufficiency.ti,ab.
20. Acute Renal Insufficiency.ti,ab.
21. Acute Kidney Failure.ti,ab.
22. Acute Renal Failure.ti,ab.
23. Respiratory Distress Syndrome.ti,ab.
24. Respiratory failure.ti,ab.
